# Oxygen Uptake Efficiency Slope and Breathing Reserve, Not Anaerobic Threshold, Discriminate Between Patients With Cardiovascular Disease Over Chronic Obstructive Pulmonary Disease

**DOI:** 10.1016/j.jchf.2015.11.003

**Published:** 2016-04

**Authors:** Anthony Barron, Darrel P. Francis, Jamil Mayet, Ralf Ewert, Anne Obst, Mark Mason, Sarah Elkin, Alun D. Hughes, Roland Wensel

**Affiliations:** a International Centre for Circulatory Health, Imperial College London and Imperial College NHS Trust, London, England; b St Mary’s Hospital, Imperial College Healthcare NHS Trust, London, England; c Department of Internal Medicine B-Cardiology, Intensive Care, Pulmonary Medicine and Infectious Diseases, University of Greifswald, Greifswald, Germany; d Harefield Hospital, Royal Brompton and Harefield NHS Foundation Trust, London, England; e Institute of Cardiovascular Sciences, University College London, London, England; f Watford General Hospital, West Herts NHS Trust, Hertfordshire, England

**Keywords:** cardiopulmonary exercise testing, chronic heart failure, exercise physiology, obstructive pulmonary disease, AT, anaerobic threshold, AUC, area under the receiver-operator characteristic curve, BNP, B-type natriuretic peptide, BR, breathing reserve, COPD, chronic obstructive pulmonary disease, CPX, cardiopulmonary exercise testing, MVV, maximal voluntary ventilation, OUES, oxygen uptake efficiency slope, ROC, receiver-operating characteristic, VO_2_, oxygen uptake

## Abstract

**Objectives:**

The study sought to compare the relative discrimination of various cardiopulmonary exercise testing (CPX) variables between cardiac and respiratory disease.

**Background:**

CPX testing is used in many cardiorespiratory diseases. However, discrimination of cardiac and respiratory dysfunction can be problematic. Anaerobic threshold (AT) and oxygen-uptake to work-rate relationship (VO_2_/WR slope) have been proposed as diagnostic of cardiac dysfunction, but multiple variables have not been compared.

**Methods:**

A total of 73 patients with chronic obstructive pulmonary disease (COPD) (n = 25), heart failure with reduced ejection fraction (HFrEF) (n = 40), or combined COPD and HFrEF (n = 8) were recruited and underwent CPX testing on a bicycle ergometer. Following a familiarization test, each patient underwent a personalized second test aiming for maximal exercise after ∼10 min. Measurements from this test were used to calculate area under the receiver-operator characteristic curve (AUC).

**Results:**

Peak VO_2_ was similar between the 2 principal groups (COPD 17.1 ± 4.6 ml/min/kg; HFrEF 16.4 ± 3.6 ml/min/kg). Breathing reserve (AUC: 0.91) and percent predicted oxygen uptake efficiency slope (OUES) (AUC: 0.87) had the greatest ability to discriminate between COPD and HFrEF. VO_2_/WR slope performed significantly worse (AUC: 0.68). VO_2_ at the AT did not discriminate (AUC for AT as percent predicted peak VO_2_: 0.56). OUES and breathing reserve remained strong discriminators when compared with an external cohort of healthy matched controls, and were comparable to B-type natriuretic peptide.

**Conclusions:**

Breathing reserve and OUES discriminate heart failure from COPD. Despite it being considered an important determinant of cardiac dysfunction, the AT could not discriminate these typical clinical populations while the VO_2_/WR slope showed poor to moderate discriminant ability. (Identifying an Ideal Cardiopulmonary Exercise Test Parameter [PVA]; NCT01162083)

Cardiopulmonary exercise testing (CPX) is recommended for the identification of the key-limiting organ in a patient presenting with exercise intolerance or dyspnea (1). Most diagnostic algorithms are similar 2, 3, 4, 5: peak oxygen uptake (VO_2_) is used to determine the extent of limitation and the combination of anaerobic threshold (AT) and breathing reserve (BR) is used to determine cause. A BR cutoff of 30% and an AT cutoff of 40% of predicted peak VO_2_ have typically been used to discriminate between respiratory and cardiac limitation respectively. However, it may be difficult to establish etiology if abnormalities in cardiac and respiratory function coexist (6). BR, measured at peak (7) or AT (8), discriminates patients with known respiratory disease from healthy adults and those with heart disease, in small, selective studies. These results have not been replicated in independent samples, these studies employed small sample sizes and participants were highly selected.

It is also unclear how best to assess change in status using serial measurements of a single patient when pulmonary and cardiac pathologies coexist, which is not uncommon. In 1 study of chronic heart failure (CHF) patients 40% had spirometry suggestive of chronic obstructive pulmonary disease (COPD) (9). Another reported that CHF was present in ∼20% of people with COPD (10).

This study aimed to establish which CPX variables showed the best ability to discriminate between respiratory and cardiac limitation in a prospective cohort of patients with COPD, heart failure with reduced ejection fraction (HFrEF), and coexisting COPD and HFrEF.

## Methods

### Recruitment

Patients with a diagnosis of HFrEF or COPD were eligible for the study. HFrEF patients were prospectively recruited from a heart failure clinic. They must have been symptomatic at some point in the past. Sequential symptomatic COPD patients were recruited from an outpatient clinic. Four patients with COPD found to have ventricular dysfunction, and 4 patients with HFrEF with obstructive spirometry and COPD features (smoking history, typical computed tomography findings, sputum production) were subsequently reclassified into a mixed group.

Exclusion criteria included: inability to perform an exercise test, significant renal impairment (estimated glomerular filtration rate <30 ml/min/1.73 m^2^), angina, recent cardiorespiratory decompensation, anemia, morbid obesity, and standard contraindications to exercise testing. All patients’ care was managed by Imperial College Healthcare NHS Trust, or Royal Brompton and Harefield Hospital NHS Trust (London, England). The study conformed to the principles of the Declaration of Helsinki and was approved by the Imperial College Healthcare NHS Trust Research Ethics Committee. Participants provided written informed consent.

Healthy controls were obtained from the SHIP (Study of Health In Pomerania) study, from which normal equations have been derived for a number of CPX variables. Study methodology has been described elsewhere (11). Briefly this population study recruited 7,008 adults with 292 persons of each sex in each 12 5-year age strata. The final sample comprised 3,300 subjects (1,589 males) 25 to 85 years of age. Of those, 1,708 individuals (834 males) volunteered for an incremental cycle exercise test from which the healthy controls were drawn. Matching was performed on a 2:1 ratio for each case; matched to gender, age within 5 years, and same body mass index category (underweight, healthy weight, overweight, obese).

### Lung function and CPX testing

All HFrEF and COPD participants underwent full lung function testing using a Spiro Air (Medisoft, Sorinnes, Belgium), and exercise testing on an ergoselect 100 ergometer (Ergoline GmbH, Bitz, Baden-Württemberg, Germany) in an air-conditioned room. Twelve-hour abstention from caffeinated products was encouraged. CPX was performed using COSMED Quark CPX System (COSMED S.r.l., Rome, Italy), calibrated before each test. Three minutes of unloaded cycling preceded a 10 W/min ramp protocol exercising to exhaustion, with breath-by-breath gas exchange data. Blood pressure was recorded every 3 min using a manual sphygmomanometer. Patients underwent a second CPX at least 2 h after this familiarization test. This was similar to the first test, with 3 min unloaded, then incremental exercise but with a ramp protocol of 6, 8, 10, 12, 15, or 20 W/min depending on the results of the initial test, to elicit exhaustion between 8 and 10 min of incremental exercise. If 6 incremental minutes were achieved on the familiarization test a 6 W second test protocol was deemed suitable; 20 min on the familiarization test led to a 20 W protocol, and so forth.

### Calculation of CPX measures

Full details of calculations and abbreviations are provided in the Online Appendix. Briefly all peak measures used the highest 20-s average. The AT was identified using unaveraged breath-by-breath data using the V-slope method (12), and corroborated using other plots. VO_2_ at AT, minute ventilation:carbon dioxide (VE/VCO_2_) ratio at AT, and end-tidal CO_2_ (P_ET_CO_2_) (mm Hg) were taken at this time point. The oxygen uptake efficiency slope (OUES) was calculated as the slope of the regression line between log_10_ minute ventilation (x-axis) and VO_2_ (y-axis). The VE/VCO_2_ slope was calculated using data until the ventilatory compensation point − slope 1, and using all exercise data, including exercise after ventilatory compensation point − slope 2. Maximal voluntary ventilation (MVV) was calculated as: 40 · forced expiratory volume in 1 s (FEV_1_). Predicted values for peak VO_2_ and OUES were generated from the SHIP study 13, 14. All other calculations were performed using standard methods. The AT was described as a percentage of predicted peak VO_2_.

A number of CPX variables were never calculated in the SHIP cohort, and so these variables were only analyzed for the 2 disease groups.

### Other measures

Echocardiography was performed using an IE33 ultrasound system (Philips, Amsterdam, the Netherlands) and B-type natriuretic peptide (BNP) was measured.

### Statistical analysis

Statistical analysis was performed using Stata version 11.1 for Windows (StataCorp LP, College Station, Texas). Normality of continuous variables was assessed using the Shapiro-Wilk test. Skewed variables were log transformed or nonparametric analyses used. A one-way analysis of variance was used to compare differences; for non-normally distributed variables a Kruskal-Wallis test was used. Categorical variables were compared with a chi-square test. Associations between FEV_1_, percent predicted FEV_1_, and the transfer coefficient corrected for Hb (K_CO_(Hb)) and CPX variables were assessed using a multivariate model including age, gender, and weight as pre-specified covariates.

The ability of each variable to discriminate between the 3 groups was assessed using the area under the receiver-operator characteristic (ROC) curve (AUC) with their 95% confidence interval. AUC ≥0.8, 0.7 to 0.8, and <0.7 were considered good, moderate, and poor respectively. Variables with good AUC were compared using the Stata roccomp command while all values were compared using the rocgold command with a Bonferroni correction. The primary analysis was a comparison of AUC in HFrEF against COPD patients (excluding mixed disease). Secondary analyses were both groups against the healthy matched controls, and the 2 disease groups including the mixed disease patients by original diagnosis. The ROC was arranged to give values ≥0.50. Variables requiring the AT to be achieved were initially only assessed in the patients who achieved the AT. To correct for the reduced number of patients achieving AT further analysis was done with peak VO_2_ substituting for VO_2_ at AT in those not achieving AT. For net reclassification improvement and integrated discrimination improvement we compared accepted cutoffs (AT <40% predicted peak VO_2_) to identify HFrEF with the optimal variable identified from ROC curve analysis. Patients were scored +1 (patient correctly reclassified to HFrEF), zero (did not change groups), or –1 (patient incorrectly reclassified as not having HFrEF).

A p value of <0.05 was considered significant throughout.

## Results

### Patient recruitment and characteristics

A total of 73 patients (58 male) were recruited; 44 had HFrEF, 15 were awaiting CRT, 16 had previously undergone CRT, and 29 had COPD. Patient characteristics of the groups are shown in Table 1. Disease groups did not significantly differ by age, but did by gender (p = 0.01) and weight (p = 0.001), and further characteristics were corrected for age, gender, and weight. Most HFrEF patients were symptomatic (5 NYHA functional class I, 30 functional class II, 9 functional class III).

**Table 1:** Patient Characteristics

	p Value Between Groups	COPD	HFrEF	Healthy Adults
Age, yrs	0.85	66.3 ± 9.9	66.7 ± 11.0	64.9 ± 9.8
Male	0.01	19 (65.6)	39 (88.6)	106 (79)
Weight, kg	0.001	70.7 ± 16.0	82.6 ± 15.3	81.0 ± 15.7
Height, cm	0.90	166.4 ± 7.5	171.8 ± 9.6	170.6 ± 7.8
Body mass index, kg/m^2^	0.89	25.4 ± 4.8	28.0 ± 4.2	27.7 ± 4.4
Diabetes	0.07	1 (3)	8 (18)	0 (0)
Current smokers	0.55	5 (17)	7 (16)	20 (15)
Hypertensive	0.06	15 (52)	20 (46)	49 (37)
Beta-blocker use	<0.001	1 (3)	38 (86)	23 (17)
ACEI/ATII-R use	<0.001	5 (17)	40 (91)	Not recorded
Hb, g/dl	0.01	14.4 ± 1.12	13.8 ± 1.35	15.4 ± 1.33
eGFR, ml/min/1.73 m^2^	0.05	83.0 ± 19.2	69.9 ± 17.9	Not recorded
BNP, pg/ml	<0.001	32.5 (14–50)	122 (85–286)	Not recorded
FVC, l	0.01	2.75 (2.29–3.06)	3.57 (2.96–4.29)	3.80 (3.01,4.29)
FVC, % predicted	0.01	88.6 ± 24.9	99.3 ± 18.7	99.0 ± 16.1
FEV_1_, l	<0.001	1.28 ± 0.48	2.50 ± 0.74	3.08 ± 0.78
FEV_1_ (% predicted)	<0.001	51.9 ± 20.3	88.5 ± 21.4	102.3 ± 15.4
FEV_1_:FVC ratio, %	<0.001	43.3 (35.8–55.9)	71.0 (62.7–76.4)	82.5 (78.0,86.0)
K_CO_, min^–1^	0.007	0.94 ± 0.38	1.15 ± 0.30	1.41 ± 0.34
Ramp protocol, W/min	0.65	10.1 ± 2.8	11.3 ± 2.9	Not recorded
LVEDV, cm^3^	<0.001	75 ± 33	161 ± 53	Not recorded
LVESV, cm^3^	<0.001	27.5 (19.4–36.7)	99.8 (68.3–145.3)	Not recorded
LVEF, %	<0.001	58.3 ± 10.5	35.3 ± 9.4	Not recorded
LVFS (%)	<0.001	27.9 ± 6.4	16.2 ± 6.9	Not recorded
LA volume, cm^3^	<0.001	42.6 (33.3–61.4)	74.6 (56.4–104.4)	Not recorded
TAPSE, mm	0.004	21 ± 4	19 ± 4	Not recorded
RV S’-wave, cm/s	<0.001	12.3 ± 3.3	10.3 ± 2.4	Not recorded

Values are mean ± SD, n (%), or median (interquartile range). The p values are between disease groups (excluding healthy controls) by analysis of variance for continuous variables and chi-square analysis for categorical variables.

ACEI = angotensin-converting enzyme inhibitor; ATII-R = angiotensin II receptor blocker; BNP = B-type natriuretic peptide; COPD = chronic obstructive pulmonary disease; eGFR = estimated glomerular filtration rate; FEV_1_ = forced expiration in 1 second; FVC = forced vital capacity; HFrEF = heart failure with reduced ejection fraction; LA = left atrial; LVEDV = left ventricular end-diastolic volume; LVEF = left ventricular ejection fraction; LVESV = left ventricular end-systolic volume; LVFS = left ventricular fractional shortening; RV = right ventricular; TAPSE = tricuspid annular plane systolic excursion.

Within the COPD category, 2 patients were categorized as mild, 12 were moderate, 10 were severe, and 3 were very severe.

The SHIP cohort matched 134 healthy controls.

### CPX results and associations with exercise capacity

Unadjusted peak VO_2_ was similar between the 2 principal groups (COPD 17.1 ± 4.6 ml/min/kg and HFrEF 16.4 ± 3.6 ml/min/kg, p = 0.48). Table 2 gives the adjusted mean values for all CPX variables within each group. 8 patients in the COPD group, 1 in the HFrEF group, 1 in the mixed group, and 2 healthy controls, did not achieve AT and were excluded from analyses on AT dependent variables only.

**Table 2:** CPX Results for a Number of Principal Variables Divided Into Disease Categories; COPD and HFrEF

	COPD (n = 25)	HFrEF (n = 40)	Healthy Adults (n = 134)
Peak VO_2_, ml/min∗†	1,356 ± 73	1,299 ± 56	1,937 ± 31
Peak VO_2_, ml/min/kg∗†	16.7 ± 0.9	16.5 ± 0.7	24.4 ± 0.4
Peak VO_2_, % predicted∗†	69.9 ± 3.4	70.6 ± 2.6	101.0 ± 1.4
AT, ml/min∗†	983 ± 60	941 ± 39	1,157 ± 22
AT, % of predicted peak VO_2_∗†	52.4 ± 2.9	51.2 ± 1.8	60.3 ± 1.0
OUES‡	2.11 ± 0.09	1.50 ± 0.07	2.27 ± 0.04
OUES/kg†§	27.0 ± 1.2	19.1 ± 0.9	28.6 ± 0.5
OUES, % predicted†§	105.6 ± 4.1	72.6 ± 3.1	102.5 ± 1.7
OUEP§	30.5 ± 1.0	33.5 ± 0.8	Not recorded
O_2_ pulse, ml/beat‡	10.7 ± 0.5	12.1 ± 0.4	13.8 ± 0.2
O_2_ pulse, % predicted∗†	77.9 ± 3.2	82.6 ± 2.5	100.0 ± 1.3
VE/VCO_2_ slope 1∗†	33.6 ± 1.1	36.0 ± 0.8	26.5 ± 0.5
VE/VCO_2_ slope 2	34.1 ± 1.9	38.6 ± 1.5	Not recorded
VE/VCO_2_ ratio nadir	35.2 ± 1.2	33.9 ± 0.9	Not recorded
VE/VCO_2_ ratio at AT∗†	38.7 ± 1.2	35.7 ± 0.8	29.0 ± 0.4
RER at peak§	0.99 ± 0.02	1.11 ± 0.02	Not recorded
P_ET_CO_2_ at AT, mm Hg∗†	34.8 ± 1.0	35.1 ± 0.7	38.7 ± 0.4
HR at peak, beats/min‡	126 ± 4	115 ± 3	141 ± 2
DP, mm Hg beats/min§	23,566 ± 1205	17,630 ± 907	28,871 ± 493
Circ power, mm Hg l/min∗†	259.3 ± 20.7	196.6 ± 15.6	398.9 ± 8.5
Peak O_2_ saturations, %§	94 ± 1	98 ± 1	Not recorded
BR at AT, %§	49.8 ± 2.8	69.5 ± 1.8	Not recorded
BR, %∗§	9.3 ± 3.0	42.6 ± 2.3	46.8 ± 1.2
VO_2_/WR slope†§	9.8 ± 0.3	8.7 ± 0.2	9.7 ± 0.1
HR/VO_2_ slope∗	0.043 ± 0.003	0.045 ± 0.002	0.042 ± 0.001
Peak work rate, W‡	85 ± 6	93 ± 5	145 ± 3
Duration, mins:s	10:32 ± 0:19	11:05 ± 0:15	N/A

Values are mean ± SE. The values represent adjusted means based on the analysis of variance model including age, gender, and weight.

AT = anaerobic threshold; BR = breathing reserve; CPX = cardiopulmonary exercise testing; DP = double product; HR = heart rate; OUES = oxygen uptake efficiency slope; P_ET_CO_2_ = end-tidal CO_2_; RER = respiratory exchange ratio; VE = minute ventilation; VO_2_ = oxygen uptake; VO_2_/WR slope = oxygen-uptake to work-rate relationship; other abbreviations as in Table 1.

∗ Significant difference between COPD and healthy adults.

† Significant difference between HFrEF and healthy adults.

‡ Significant differences between all groups.

§ Significant difference between COPD and HFrEF.

Among the patients, peak VO_2_ correlated with estimated glomerular filtration rate (r = 0.30, p = 0.003) and Log_10_BNP (r = –0.35, p = 0.001) but not with hemoglobin or sodium. Fourteen individuals had atrial fibrillation or flutter but there was no significant difference in peak VO_2_ compared to sinus rhythm (p = 0.24).

### Associations between spirometry and CPX in HFrEF

On multivariate regression analysis peak VO_2_ did not relate to FEV_1_ in patients with HFrEF (p = 0.20). Only breathing reserve at AT (p = 0.03) and peak (p = 0.01), and peak minute ventilation (p = 0.04) related to FEV_1_, while only O_2_-pulse related to K_CO_(Hb) with borderline significance (p = 0.047).

### Differences in variables between groups

**Figure 1:**
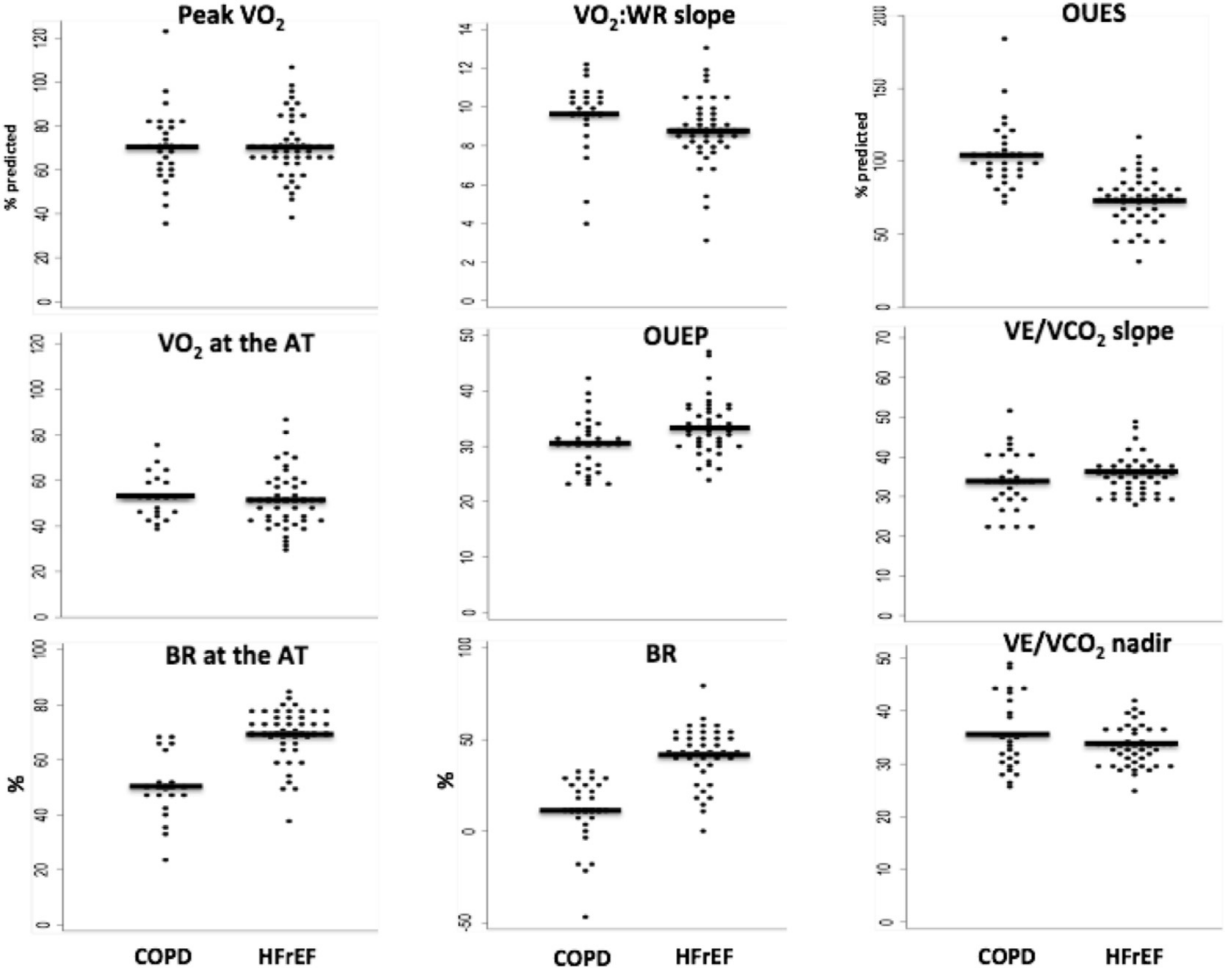
Dotplots for 9 CPX Variables in Patients With COPD and HFrEF Dotplots for 9 cardiopulmonary exercise testing variables in patients with chronic obstructive pulmonary disease (COPD) and heart failure with reduced ejection fraction (HFrEF). Each dot represents an individual with mean values represented by black lines. AT = anaerobic threshold; BR = breathing rate; VO_2_ = oxygen uptake; VO_2_:WR slope = oxygen-uptake to work-rate relationship.

A comparison of results across groups is shown in Table 2, and between the 2 disease groups in Figure 1. All measures of peak VO_2_, the AT, the VE/VCO_2_-slope and ratio at AT, end-tidal CO_2_, and circulatory power all showed significant differences between healthy controls and each disease group, but not between the disease groups. OUES, OUES/kg, and percent predicted OUES differed significantly between COPD and HFrEF, and between HFrEF and healthy controls. OUES also differed between COPD and healthy controls but not when corrected for weight or percent predicted. The unadjusted O_2_-pulse differed between all groups, and was significantly higher in the HFrEF than the COPD group but the 2 disease states did not differ as percent predicted. Double product differed between all groups, with lowest values in the HFrEF group, and highest in healthy controls. Breathing reserve at the AT was significantly lower in the COPD compared to the HFrEF group and at peak was significantly lower in the COPD compared with both other groups. VO_2_ to work-rate relationship was significantly lower in patients with HFrEF compared to the other groups. OUEP was significantly lower in the COPD compared to the HFrEF group.

### Receiver-operating characteristic curve analysis and reclassification indices

Table 3 shows comparisons of the discriminant abilities of the variables, quantified as AUCs. Variables with good discrimination between COPD and HFrEF were breathing reserve (AUC: 0.91), breathing reserve at AT (AUC: 0.89), OUES/kg (AUC: 0.84), and percent predicted OUES (AUC: 0.87); none of these AUC values differed significantly. Double product and O_2_ pulse were moderate discriminators but not significantly different from breathing reserve (the discriminator with the greatest AUC) after a Bonferroni correction. Other variables were either significantly worse discriminators or did not discriminate at all.

**Table 3:** AUC for a Number of CPX Variables

	HFrEF Versus COPD (Excluding Mixed)	HFrEF Versus Healthy Adults	COPD Versus Healthy Adults
BR, %	0.91 (0.84–0.98)	0.58 (0.49–0.68)	0.96 (0.94–0.99)
Breathing reserve at AT, %	0.89 (0.80–0.98)	—	—
OUES, % predicted	0.87 (0.79–0.96)	0.89 (0.82–0.95)	0.52 (0.39–0.66)
OUES/kg	0.84 (0.75–0.93)	0.89 (0.84–0.95)	0.59 (0.45–0.72)
O_2_ pulse, ml/beat	0.80 (0.69–0.91)	0.61 (0.51–0.71)	0.89 (0.84–0.95)
Peak oxygen saturations, %	0.79 (0.68–0.90)	—	—
DP, mm Hg beats/min	0.78 (0.67–0.90)	0.91 (0.86–0.95)	0.75 (0.65–0.84)
RER at peak	0.75 (0.62–0.88)	—	—
VO_2_/WR slope	0.68 (0.54–0.83)	0.70 (0.60–0.81)	0.52 (0.38–0.66)
HR at peak, beats/min	0.68 (0.55–0.81)	0.80 (0.73–0.88)	0.70 (0.60–0.80)
Peak VO_2_, ml/min	0.66 (0.52–0.79)	0.82 (0.75–0.88)	0.90 (0.84–0.95)
OUEP	0.65 (0.51–0.80)	—	—
OUES	0.65 (0.51–0.78)	0.83 (0.76–0.90)	0.71 (0.60–0.83)
AT, ml/min	0.65 (0.49–0.80)	0.67 (0.58–0.76)	0.79 (0.69–0.89)
VE/VCO_2_ slope 2	0.64 (0.48–0.79)	—	—
HR/VO_2_ slope	0.63 (0.49–0.77)	0.50 (0.39–0.62)	0.65 (0.54–0.76)
VE/VCO_2_ ratio AT	0.59 (0.42–0.77)	0.85 (0.79–0.91)	0.88 (0.80–0.97)
VE/VCO_2_ slope 1	0.57 (0.42–0.73)	0.92 (0.88–0.96)	0.77 (0.65–0.90)
AT, % of predicted peak VO_2_	0.56 (0.40–0.72)	0.72 (0.62–0.82)	0.68 (0.54–0.83)
O_2_ pulse, % predicted	0.56 (0.41–0.71)	0.79 (0.70–0.88)	0.82 (0.72–0.92)
VE/VCO_2_ ratio nadir	0.54 (0.38–0.70)	—	—
Peak VO_2_, ml/min/kg	0.54 (0.38–0.69)	0.89 (0.83–0.94)	0.83 (0.74–0.91)
Circ power, mm Hg ml/min	0.53 (0.37–0.68)	0.90 (0.86–0.95)	0.88 (0.81–0.94)
Peak VO_2_, % predicted	0.51 (0.36–0.66)	0.91 (0.86–0.96)	0.90 (0.82–0.98)
P_ET_CO_2_ at AT, mm Hg	0.51 (0.32–0.69)	0.73 (0.64–0.82)	0.71 (0.58–0.85)

Values are area under the curve (AUC) calculated following 3 receiver-operating characteristic curve analyses: the primary analysis was patients with heart failure versus patients with COPD (excluding mixed disease); patients with heart failure versus healthy matched controls from the SHIP cohort; and patients with COPD versus healthy matched controls from the SHIP (Study of Health In Pomerania) cohort. The AUC does not indicate the direction of the discrimination. The variables are ordered by the AUC for the primary analysis and grouped as good (AUC: >0.8, top 4 variables), moderate (AUC: >0.70, next 4 variables), and poor discrimination (AUC: ≤0.70, remaining variables).

Abbreviations as in Tables 1 and 2.

Variables with good discrimination between COPD and healthy controls were breathing reserve, peak VO_2_, VE/VCO_2_ at AT, O_2_-pulse, and circulatory power.

Variables with good discrimination between HFrEF and healthy controls were OUES, double product, peak VO_2_, circulatory power, VE/VCO_2_ slope, and VO_2_ at AT.

Including patients with mixed disease under their primary diagnosis worsened discrimination marginally (Online Table 1).

To ensure that patients not achieving AT were not influencing its power to detect a difference in groups, peak VO_2_ was substituted for the AT in these patients. The AUC for the VO_2_ at AT in ml/min was 0.60 and 0.57 as percent predicted peak VO_2_, both similar to the values seen when those not achieving the AT were excluded.

**Figure 2:**
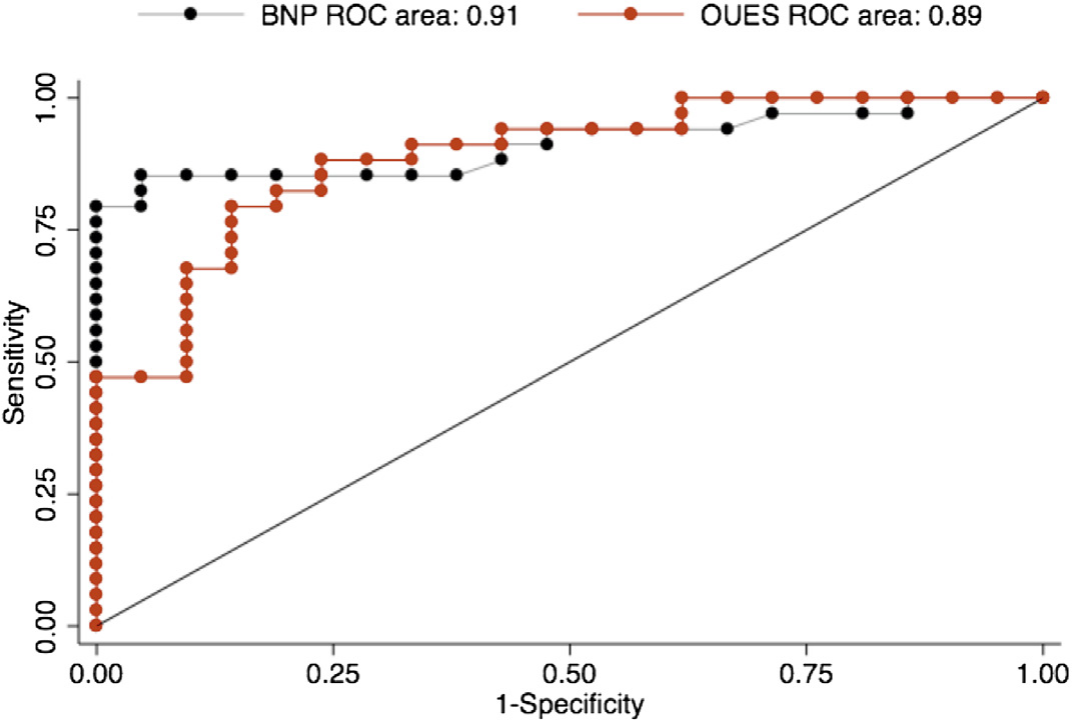
Comparison of the Area Under the ROC Curve for BNP and Percent Predicted OUES BNP = B-type natriuretic peptide; OUES = oxygen uptake efficiency slope; ROC = receiver-operating characteristic.

BNP measurements were obtained in 55 of 65 patients and showed an AUC of 0.91, which was not significantly different from BR or percent predicted OUES (Figure 2). The addition of BNP to a logistic model including percent predicted OUES improved the AUC nonsignificantly from 0.90 to 0.95 (p = 0.07). The further addition of BR again nonsignificantly increased the AUC to 0.98 (p = 0.10).

Optimal cutoffs were identified for percent predicted OUES and BR. A threshold of 89.2% predicted OUES showed sensitivity of 80% and specificity of 85% to predict HFrEF, correctly classifying 54 of 65 patients without mixed disease. A threshold of 33.6% for BR showed sensitivity of 80% and specificity of 100% to predict respiratory disease, correctly classifying 56 of 65 patients without mixed disease.

Using a previously determined algorithm (5) that used the cutoffs for AT of 40% predicted peak VO_2_, and BR of 30%, 26 of 65 patients were correctly classified. Net reclassification improvement for percent predicted OUES over AT showed an improvement of 74.0% (p < 0.001), with an integrated discrimination improvement of 29.6% (p < 0.001).

## Discussion

Among CPX variables OUES and breathing reserve displayed the greatest ability to discriminate between HFrEF and COPD, and were the only CPX discriminators with AUC >0.8. This discriminant ability was similar to that seen with BNP. OUES also strongly discriminated HFrEF from healthy adults, while BR discriminated COPD from healthy adults.

**Figure 3:**
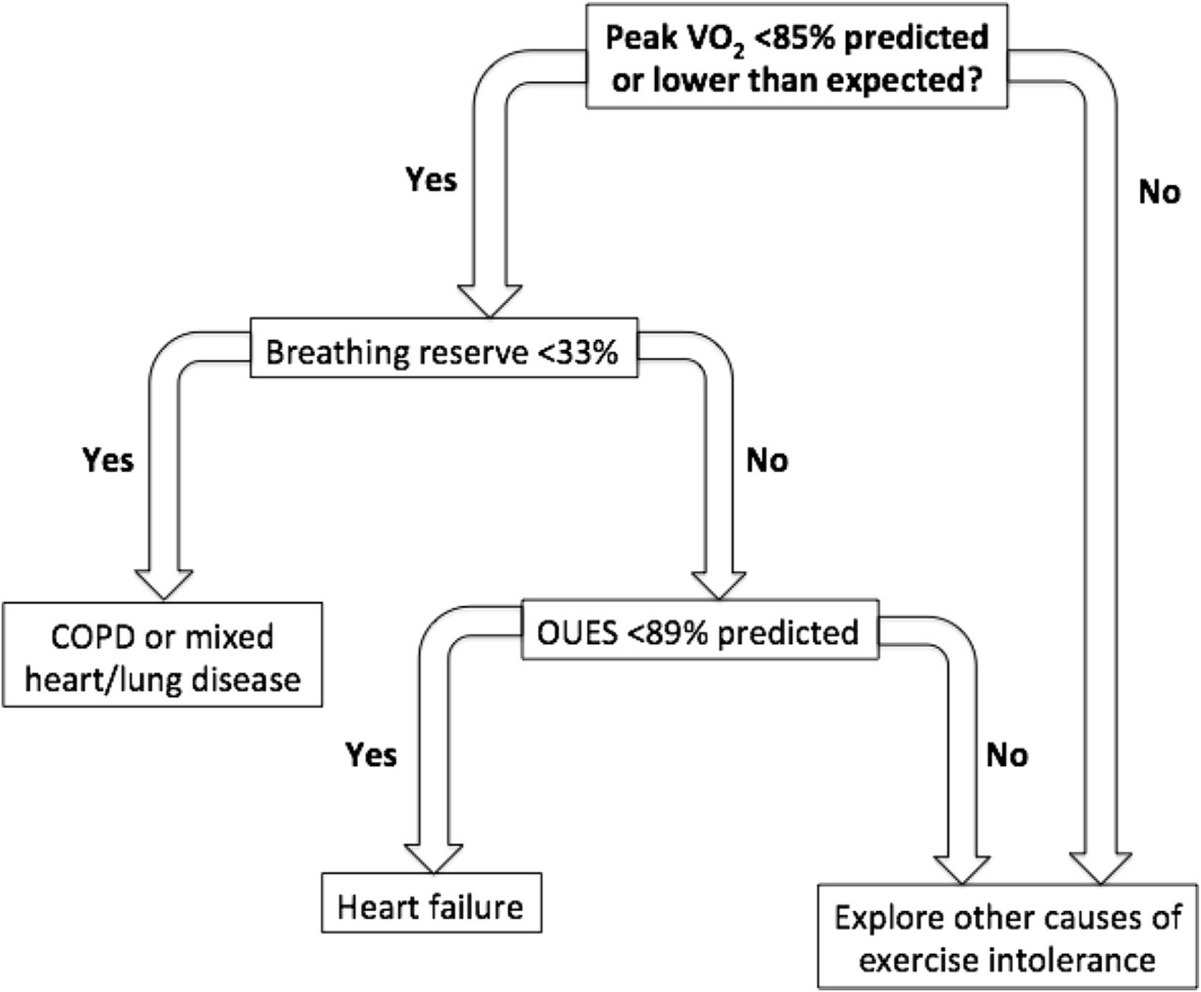
Proposed Algorithm for the Identification of Respiratory or Cardiac Disease in a Patient With Exercise Limitation Breathing reserve (%) is calculated as 100× (maximum voluntary ventilation [MVV] – peak minute ventilation)/MVV, where MVV was calculated as: 40 · FEV_1_ at rest. The percent predicted oxygen uptake efficiency slope (OUES) was calculated using data from Barron et al. (14). COPD = chronic obstructive pulmonary disease; VO_2_ = oxygen uptake.

A potential algorithm to help distinguish patients based on these variables is shown in Figure 3.

Peak VO_2_, the most widely known CPX variable, had no capacity to discriminate between cardiac and pulmonary causes of exercise limitation (AUC: ∼0.50); both diseases depressed peak VO_2_. Importantly this similarity of peak VO_2_ between our 2 principal groups allowed us to compare the ability of other measures to discriminate between cardiac and lung disease without concerns that observed differences merely related to differences in peak VO_2_.

OUES, BR, and VO_2_ at AT are described individually in detail subsequently. Select other variables are described now briefly.

VO_2_/WR slope had significantly lower values in patients with HFrEF but was only moderately discriminant, while in COPD it behaved similarly to healthy adults. A previous study showing lower slopes in cardiovascular disease compared with healthy controls (15) had shorter exercise times among the patients, which may influence its value (16). In our current study individualized protocols resulted in similar, and recommended, exercise times (1), suggesting that cardiovascular limitation may lower the slope even with optimal exercise duration.

The O_2_-pulse, a surrogate for stroke volume, was unexpectedly higher in HFrEF, compared to COPD, although this difference was not seen when corrected using predictive equations. We believe that high beta-blockade use within the HFrEF group led to lower heart rates, greater filling times, and therefore higher stroke volumes. Second, the O_2_-pulse is dependent on arteriovenous oxygen content difference, often reduced in COPD patients with lower arterial saturations and higher peak venous saturations. The predicted O_2_-pulse was unsurprisingly significantly higher in healthy controls compared to both disease groups.

All measurements of the VE/VCO_2_ relationship failed to discriminate the disease groups, but were significantly higher than healthy controls. In patients with HFrEF they are abnormal due to hyperventilation and *perfusion* to ventilation mismatching (17). In COPD a number of causes lead to an abnormal VE/VCO_2_ relationship including mismatching of *ventilation* to perfusion.

### Oxygen uptake efficiency slope

The OUES, largely effort independent, is calculated as the slope of the semilog relationship between O_2_ and minute ventilation (18). OUES appears to be unaffected by COPD. Our group has previously found, within a large retrospective heart failure cohort, that patients with low percent predicted FEV_1_ have lower peak VO_2_ but not OUES (19). In the current study OUES did not relate to FEV_1_ or K_CO_(Hb).

OUES was significantly lower in HFrEF than COPD, despite similar exercise capacities, and healthy controls, and on ROC curve analysis OUES, when weight adjusted or as a percent predicted, was a good diagnostic indicator of HFrEF with similar power to discriminate these 2 common causes of breathlessness as BNP. Importantly, OUES, when corrected, appears unaffected by COPD. Given the small numbers of patients within the mixed group further evaluation of the role of the OUES in patients with mixed disease would be beneficial.

Why did OUES differ? Patients with heart failure typically have an abnormal peak VO_2_ but may still ventilate to high levels. A considerable portion of exercise occurs where the VE and VO_2_ relationships are decoupled because of anaerobic metabolism and hyperventilation. This results in increasing levels of alveolar pO_2_, worse ventilatory efficiency towards peak exercise, and a “flattened” relationship 19, 20, 21.

In contrast COPD patients behave similar to healthy adults failing to progress to maximal exercise, and are less anerobic at peak exercise. Furthermore, due to the increasing ventilatory constraint during exercise the increase in alveolar pO_2_ as a result of anaerobic metabolism is less pronounced, and alveolar pO_2_ may actually fall toward peak, thereby rendering ventilation more efficient (22), offsetting inefficient mechanisms such as ventilation:perfusion mismatch. Therefore, the VO_2_/log_10_VE curve may be “shifted” rightward (higher log_10_VE for any given VO_2_) but the curve’s gradient itself is unchanged. This hypothesis may also explain why our mixed cohort had OUES values close to predicted.

### Breathing reserve

Breathing reserve has long been suggested as a discriminator of respiratory limitation 3, 4, 5, 8. BR at AT has been proposed to reduce the influence of voluntarily cessation of exercise (8). Both BR at AT and peak showed good discriminatory power. However 32% of our COPD patients did not achieve AT, similar to the study advocating the BR at AT (40%), limiting its widespread applicability. In contrast BR at peak and the OUES are measurable in all.

The BR at peak is useful in identifying the principal physiology limiting exercise. However, unlike OUES, the magnitude is unlikely to be useful when measuring disease severity, so its role in serial studies in 1 patient may be limited.

BR is low in COPD because although in both HFrEF and COPD peak minute ventilation is reduced, in COPD patients the maximum voluntary ventilation (MVV, a function of FEV_1_) is typically much lower, leading to a smaller gap between peak minute ventilation and MVV; the BR. Patients with mixed disease had low BR values suggesting this variable may not be able to distinguish between those with COPD alone or those with mixed disease.

### Anaerobic threshold

In previous CPX algorithms a reduced AT would identify heart failure 4, 5, yet evidence supporting its role is scarce. In HFrEF VO_2_ at AT is reduced (23) and superior to peak VO_2_ at predicting prognosis (24). Nery et al. (7), showed VO_2_ at AT in patients with mitral valve disease was lower than patients with COPD and healthy controls; however the numbers were small with significant differences in gender and age between groups. These studies are the foundation of what has become a firmly held belief—namely that VO_2_ at AT reflects cardiac function. Very few CPX studies performed on patients with COPD report the VO_2_ at AT however Medoff et al. (8) found no difference between COPD and heart failure patients with similar exercise capacities, consistent with our findings. We showed that VO_2_ at AT (as a percent predicted peak VO_2_) has poor discriminant ability between the disease groups, and only showed moderate discrimination between healthy adults and the 2 groups. We suggest that this variable is critically determined by muscle function and any chronic process that impairs muscular function will reduce the AT.

There may be a concern that the number of patients without a measured AT influenced our results. Our results are similar to other studies in terms of numbers of COPD patients failing to achieve AT (8), and we believe representative of patients. In all cases we believe AT had not been achieved rather than being unidentifiable. The failure to attain AT (9 of 29 COPD patients compared with 1 of 44 HFrEF patients) would appear to display good specificity, but poor sensitivity, to diagnose COPD over HFrEF. The substitution of peak VO_2_ for the VO_2_ at AT in patients failing to achieve AT allowed for all patients to be involved in the analysis of the AT variables but did not significantly change the ROC results.

### Study limitations

Some of the patients within the study population were not as symptomatic as expected, with 16% achieving 85% predicted peak VO_2_. We hope to inspire future studies addressing more severely affected patients, for example those awaiting heart or lung transplant. We also made an assumption that principal pathophysiology was limiting exercise capacity. It is possible that patients were limited by musculoskeletal problems or adiposity. However, this should attenuate between-group differences.

Prevalence of mixed disease was lower than anticipated—only 9% of HFrEF and 14% of COPD patients. Consequently it was not feasible to analyze mixed disease as a separate group. We performed sensitivity analyses by comparing inclusion with exclusion of these individuals and found it had minimal effects on estimates of discrimination. However, future studies including patients with confirmed coexistent COPD and HFrEF would be valuable.

When comparing the mean values for CPX variables between the groups, corrections were not made for multiple comparisons, and so with large numbers of statistical comparisons it is possible that some of the significant differences are due to chance. Whilst comparing AUC values for the primary analysis on ROC curve analysis Bonferroni corrections were used.

Identification of the AT can be challenging; it is arguable that misidentification might have led to its results. Our protocol was designed for optimal exercise duration, with frequent, small workload increases (2 to 5 W increments every 12 to 20 s), which should aid identification. If despite this, misidentification still occurred it is arguably an inherent weakness of the AT, and does not negate our findings.

Medication use differed between groups, most importantly beta-blockade. Predictive equations for O_2_-pulse and OUES accounted for beta-blocker use, to minimize between-group differences.

COPD was chosen as the archetypal respiratory, and HFrEF as the archetypal cardiac diseases. However the physiological abnormalities differ significantly from other respiratory diseases such as parenchymal lung disease, and other cardiac disease states such as heart failure with preserved ejection fraction or valvular disease. Therefore our results only apply to COPD and HFrEF. Further work would be needed to show that OUES and BR discriminate a fuller spectrum of cardiorespiratory disorders.

## Conclusions

OUES and breathing reserve were the best CPX variables at discriminating HFrEF from COPD, and similar to BNP. VO_2_ at AT did not discriminate HFrEF patients from COPD. In a patient with exercise limitation, BR, and OUES could be used to identify the principal pathophysiology.

Perspectives **COMPETENCY IN MEDICAL KNOWLEDGE:** The identification of markers of heart failure severity help health care professionals to target appropriate individuals for optimal disease management including advanced heart failure therapies such as transplantation and mechanical circulatory support. CPX testing already has a central role; here we show which measured variables are most, and least, affected by heart failure, and another common condition COPD, to give physicians greater understanding of the severity of their patients’ conditions.

**TRANSLATIONAL OUTLOOK:** Heart failure and COPD commonly coexist, yet little research has been done into the exercise physiology of patients with both conditions. We show how 2 variables, OUES and BR, are good discriminators of these 2 conditions, and hope to inspire future researchers to further explore exercise pathophysiology in patients with coexistent cardiac and respiratory conditions. We also hope to promote further research into a potential role for the OUES in patient selection for advanced heart failure therapies.

## References

[1] Balady G.J., Arena R., Sietsema K. (2010). Clinician's Guide to cardiopulmonary exercise testing in adults: a scientific statement from the American Heart Association. Circulation.

[2] Neuberg G.W., Friedman S.H., Weiss M.B., Herman M.V. (1988). Cardiopulmonary exercise testing. The clinical value of gas exchange data. Arch Intern Med.

[3] Eschenbacher W.L., Mannina A. (1990). An algorithm for the interpretation of cardiopulmonary exercise tests. Chest.

[4] Milani R.V., Lavie C.J., Mehra M.R. (2004). Cardiopulmonary exercise testing: how do we differentiate the cause of dyspnea?. Circulation.

[5] Wasserman K., Hansen J.E., Sue D.Y., Stringer W.W., Whipp B.J. (2005).

[6] Wasserman K., Whipp B.J. (1975). Exercise physiology in health and disease. Am Rev Respir Dis.

[7] Nery L.E., Wasserman K., French W., Oren A., Davis J.A. (1983). Contrasting cardiovascular and respiratory responses to exercise in mitral valve and chronic obstructive pulmonary diseases. Chest.

[8] Medoff B.D., Oelberg D.A., Kanarek D.J., Systrom D.M. (1998). Breathing reserve at the lactate threshold to differentiate a pulmonary mechanical from cardiovascular limit to exercise. Chest.

[9] Mascarenhas J., Lourenco P., Lopes R., Azevedo A., Bettencourt P. (2008). Chronic obstructive pulmonary disease in heart failure. Prevalence, therapeutic and prognostic implications. Am Heart J.

[10] Rutten F.H., Cramer M.J., Lammers J.W., Grobbee D.E., Hoes A.W. (2006). Heart failure and chronic obstructive pulmonary disease: an ignored combination?. Eur J Heart Fail.

[11] Volzke H., Alte D., Schmidt C.O. (2011). Cohort profile: The study of health in Pomerania. Int J Epidemeiol.

[12] Beaver W.L., Wasserman K., Whipp B.J. (1986). A new method for detecting anaerobic threshold by gas exchange. J Appl Phyisiol.

[13] Gläser S., Koch B., Ittermann T. (2010). Influence of age, sex, body size, smoking, and beta blockade on key gas exchange exercise parameters in an adult population. Eur J Cardiovasc Prev Rehabil.

[14] Barron A.J., Dhutia N.M., Gläser S. (2015). Physiology of oxygen uptake kinetics: Insights from incremental cardiopulmonary exercise testing in the Study of Health in Pomerania. IJC Metabolic and Endocrine.

[15] Hansen J.E., Sue D.Y., Oren A., Wasserman K. (1987). Relation of oxygen uptake to work rate in normal men and men with circulatory disorders. Am J Cardiol.

[16] Agostoni P., Bianchi M., Moraschi A. (2005). Work-rate affects cardiopulmonary exercise test results in heart failure. Eur J Heart Fail.

[17] Wensel R., Georgiadou P., Francis D.P. (2004). Differential contribution of dead space ventilation and low arterial pCO_2_ to exercise hyperpnea in patients with chronic heart failure secondary to ischemic or idiopathic dilated cardiomyopathy. Am J Cardiol.

[18] Baba R., Nagashima M., Goto M. (1996). Oxygen uptake efficiency slope: a new index of cardiorespiratory functional reserve derived from the relation between oxygen uptake and minute ventilation during incremental exercise. J Am Coll Cardiol.

[19] Barron A.J., Medlow K.I., Giannoni A. (2010). Reduced confounding by impaired ventilatory function with oxygen uptake efficiency slope and VE/VCO_2_-slope rather than peak oxygen consumption to assess exercise physiology in suspected heart failure. Congest Heart Fail.

[20] Scott A.C., Wensel R., Davos C.H. (2003). Skeletal muscle reflex in heart failure patients: role of hydrogen. Circulation.

[21] Sullivan M.J., Higginbotham M.B., Cobb F.R. (1988). Increased exercise ventilation in patients with chronic heart failure: intact ventilatory control despite hemodynamic and pulmonary abnormalities. Circulation.

[22] Wasserman K., Van Kessel A.L., Burton G.G. (1967). Interaction of physiological mechanisms during exercise. J Appl Physiol.

[23] Weber K.T., Kinasewitz G.T., Janicki J.S., Fishman A.P. (1982). Oxygen utilization and ventilation during exercise in patients with chronic cardiac failure. Circulation.

[24] Gitt A.K., Wasserman K., Kilkowski C. (2002). Exercise anaerobic threshold and ventilatory efficiency identify heart failure patients for high risk of early death. Circulation.

