# Supplementary material for: Oxygen Uptake Efficiency Slope and Breathing Reserve, Not Anaerobic Threshold, Discriminate Between Patients With Cardiovascular Disease Over Chronic Obstructive Pulmonary Disease
Source: JACC Heart Fail. 2016 Apr;4(4):252–61. doi: 10.1016/j.jchf.2015.11.003 (PMC4820007; doi:10.1016/j.jchf.2015.11.003)
Supplement: Online Appendix and Online Table 1 [file mmc1.docx]

# Online Supplement

## Measurement of CPX variables

Peak VO_2_ was defined as the highest 20s average oxygen uptake during the last 60s of exercise and the first 10s of recovery. This was calculated as an absolute value (ml/min), a weight-adjusted value (ml/min kg) and as a percentage of predicted^[[1]](#endnote-1)^. Peak minute ventilation (VE) and O_2_ pulse were similarly defined as the highest 20s average during the last 60s of exercise and first 10s of recovery.

The anaerobic threshold (AT) was identified from a single breath using the V-Slope method^[[2]](#endnote-2)^ and corroborated using data from the plots of ventilatory equivalents, the respiratory exchange ratio (RER) and the end-tidal CO_2_ (P_ET_CO_2_) and O_2_ values. All measures at the AT (oxygen uptake at the AT in ml/min and percentage of predicted peak VO_2_, VE/VCO_2_ ratio at the AT, and P_ET_CO_2_ (mmHg)) were taken at this time point without averaging. The Oxygen Uptake Efficiency Slope (OUES) was calculated as the slope of the regression line between log_10_ minute ventilation (x-axis) and oxygen uptake (y-axis) using all data throughout incremental exercise (as units of L/ 10-fold increase in VE). The heart rate/ oxygen uptake (HR/VO_2_) slope (beats/ml/min) was calculated as the slope of the regression line between oxygen uptake (x-axis) and heart rate (y-axis) using all data points throughout incremental exercise. The y-axis intercept of the regression line (HR/VO_2_ intercept) was also estimated. The oxygen uptake/ work rate (VO_2_/WR) slope (ml/min/W) was calculated as the slope of the regression line between work rate (x-axis) and oxygen uptake (y-axis) excluding data preceding 10W of exercise. The VE/VCO_2_ slope was calculated in two ways, firstly Slope 1 included all exercise data up to the ventilatory compensation point (VCP) and secondly Slope 2 included all data points from onset of exercise to peak exercise (i.e. including data post-VCP). Both of these slope measurements were analysed separately as individual variables for reproducibility. The relationship between minute ventilation and CO_2_ production was also expressed as the VE/VCO_2_ ratio at nadir, which was the lowest instantaneous measurement of the ratio throughout unloaded or incremental exercise. The Oxygen Uptake Efficiency Plateau was defined as the highest 90 second average of the VO_2_/VE ratio throughout unloaded and incremental exercise^[[3]](#endnote-3)^. Breathing reserve (%) was calculated as 100 x (MVV – VE) / MVV, where VE was either the minute ventilation at peak exercise or at the AT, and MVV was the maximum voluntary ventilation calculated as 40 x FEV_1_ (obtained from formal full lung function testing). The double product (DP) was calculated as the product of the heart rate at peak exercise and the systolic blood pressure at peak exercise (mmHg bpm). Peak circulatory power was calculated as the product of the systolic blood pressure at peak exercise and the peak VO_2_ (mmHg ml/min). Predicted values for peak VO_2_, O_2_-pulse and OUES were generated from predictive equations derived from the Study of Health in Pomerania (SHIP)^[[4]](#endnote-4),^^[[5]](#endnote-5)^ and are shown below for peak VO_2_, O_2_-pulse and OUES.

**Peak VO_2_:**

Males: -69+(1.48*age)+(14.02*Height)+(7.44*Weight)-(233.72*cs)-(0.2256*age*age)

Females: -588-(11.33*age)+(9.13*Height)+(26.88*Weight)-(0.12*(Weight*Weight))

**O_2_-pulse:**

Males: -0.7-(0.044*age)+(0.064*Height)+(0.086*Weight)-(0.62*cs)+ (1.73*bb)

Females: -3.7-(0.004*age) + (0.056*Height)+(0.075*Weight)+ (0.42*bb)

**OUES**

Males: 907.7-(11.51*age) +(5.67*Height) +(8.62*Weight)-(49.99*bb)-(214.53*cs) +(172.97*FEV_1_)

Females: -182.4-(8.89*age) +(10.12*Height) +(10.51*Weight)-(117.65*bb)-(21.45*cs) +(40.31*FEV_1_)

Where: Height (cm), weight (kg), bb (beta-blocker use=1, no use =0), cs (current smoker=1, non-smoker/ex-smoker =0), FEV_1_ (L).

1. Wasserman K HJ, Sue DY, Stringer WW, ,Whipp BJ. Principles of exercise testing and interpretation. Lippincott Williams & Wilkins; 2005. [↑](#endnote-ref-1)
2. Beaver WL, Wasserman K, Whipp BJ. A new method for detecting anaerobic threshold by gas exchange. Journal of applied physiology: respiratory, environmental and exercise physiology. 1986;60:2020-2027 [↑](#endnote-ref-2)
3. Sun XG, Hansen JE, Stringer WW. Oxygen uptake efficiency plateau best predicts early death in heart failure. Chest. 2012;141:1284-1294 [↑](#endnote-ref-3)
4. Gläser S, Koch B, Ittermann T, Schäper C, Dörr M, Felix SB, Völzke H, Ewert R, Hansen JE. Influence of age, sex, body size, smoking, and beta blockade on key gas exchange exercise parameters in an adult population. Eur J Cardiovasc Prev Rehabil. 2010 Aug;17(4):469-76 [↑](#endnote-ref-4)
5. Barron AJ, Dhutia NM, Gläser S, Koch B, Ewert R, Obst A, Dörr M, Völzke H, Francis DP, Wensel R. Physiology of oxygen uptake kinetics: Insights from incremental cardiopulmonary exercise testing in the Study of Health in Pomerania. IJC Metabolic and Endocrine. 2015;7:3-9

   **Supplemental Table 1: Area under curve (AUC) for a number of CPX variables, comparing the primary analysis with a secondary analysis of 2 disease groups with all patients included by primary diagnosis (no excluded patients with mixed disease)**

   |  |  | HFrEF vs COPD (excluding mixed) | HFrEF vs COPD (by primary diagnosis) |
   | --- | --- | --- | --- |
   | Breathing reserve (%) |  | 0.91 (0.84-0.98) | 0.89 (0.83-0.95) |
   | Breathing reserve at AT (%) |  | 0.89 (0.80-0.98) | 0.87 (0.79-0.95) |
   | OUES (% predicted) |  | 0.87 (0.79-0.96) | 0.86 (0.78-0.93) |
   | OUES/kg |  | 0.84 (0.75-0.93) | 0.80 (0.71-0.89) |
   |  |  |  |  |
   | O_2_ Pulse (ml/beat) |  | 0.80 (0.69-0.91) | 0.67 (0.57-0.78) |
   | Peak oxygen saturations (%) |  | 0.79 (0.68-0.90) | 0.74 (0.63-0.85) |
   | DP (mmHg bpm) |  | 0.78 (0.67-0.90) | 0.64 (0.52-0.75) |
   | RER at peak |  | 0.75 (0.62-0.88) | 0.77 (0.66-0.88) |
   |  |  |  |  |
   | VO_2_/WR Slope |  | 0.68 (0.54-0.83) | 0.70 (0.57-0.82) |
   | HR at peak (bpm) |  | 0.68 (0.55-0.81) | 0.52 (0.40-0.63) |
   | Peak VO_2_ (ml/min) |  | 0.66 (0.52-0.79) | 0.62 (0.51-0.74) |
   | OUEP |  | 0.65 (0.51-0.80) | 0.65 (0.52-0.78) |
   | OUES |  | 0.65 (0.51-0.78) | 0.65 (0.54-0.77) |
   | AT (ml/min) |  | 0.65 (0.49-0.80) | 0.58 (0.44-0.72) |
   | VE/VCO_2_ slope 2 |  | 0.64 (0.48-0.79) | 0.64 (0.51-0.77) |
   | HR/VO_2_ Slope |  | 0.63 (0.49-0.77) | 0.51 (0.39-0.63) |
   | VE/VCO_2_ ratio AT |  | 0.59 (0.42-0.77) | 0.54 (0.38-0.70) |
   | VE/VCO_2_ slope 1 |  | 0.57 (0.42-0.73) | 0.57 (0.43-0.71) |
   | AT (% of pred peak VO_2_) |  | 0.56 (0.40-0.72) | 0.58 (0.44-0.71) |
   | O_2_ Pulse (% predicted) |  | 0.56 (0.41-0.71) | 0.52 (0.40-0.64) |
   | VE/VCO_2_ ratio nadir |  | 0.54 (0.38-0.70) | 0.53 (0.39-0.67) |
   | Peak VO_2_ (ml/min/kg) |  | 0.54 (0.38-0.69) | 0.50 (0.37-0.63) |
   | Circ power (mmHg ml/min) |  | 0.53 (0.37-0.68) | 0.51 (0.38-0.63) |
   | Peak VO_2_ (% predicted) |  | 0.51 (0.36-0.66) | 0.51 (0.38-0.64) |
   | P_ET_CO_2_ at AT (mmHg) |  | 0.51 (0.32-0.69) | 0.56 (0.39-0.72) |

   AUC calculated following 2 ROC curve analyses: the primary analysis of patients with only heart failure versus only COPD (n=65; excluding those with mixed disease); and secondary analysis of all patients with heart failure versus COPD (n=73; including those with mixed disease). The AUC does not indicate the direction of the discrimination. The variables are ordered by the AUC for the primary analysis and grouped as good (AUC>0.8, top 4 variables), moderate (AUC>0.70, next 4 variables) and poor discrimination (AUC≤0.70, remaining variables). [↑](#endnote-ref-5)
